# Efficacy of psychological treatment for headache disorder: a systematic review and meta-analysis

**DOI:** 10.1186/s10194-019-0965-4

**Published:** 2019-02-14

**Authors:** Hye Jeong Lee, Jin Hyeok Lee, Eun Young Cho, Sun Mi Kim, Seoyoung Yoon

**Affiliations:** 1 Department of Psychiatry, Catholic university of Daegu, School of Medicine, 33, Duryugongwon-ro 17-gil, Nam-gu, Daegu, Republic of Korea; 2 Department of Biostatistics, Korea University Graduate School, Seoul, South Korea; 3 Department of Psychiatry, College of Medicine, Chung-Ang University, Seoul, South Korea

**Keywords:** Headache disorders, Behavior therapy, Cognitive therapy, Mindfulness, Biofeedback, Meta-analysis, Abbreviations, BFT biofeedback, CBT Cognitive behavior therapy, MBT mindfulness-based treatment; RCTs, randomized controlled trials; MIDAS, migraine disability assessment, NICE National Institute for Health and Care Excellence, SD standard deviations; RR, relative risk

## Abstract

**Background:**

Headache disorder is not only a common complaint but also a global burden. Pharmacotherapeutic and non-pharmacotherapeutic approaches have been developed for its treatment and prophylaxis. The present study included a systematic review of psychological treatments for primary headache disorder accessible in Korea.

**Methods:**

We included English and Korean articles from EMBASE, MEDLINE, Cochrane library database, SCOPUS, ScienceDirect, Web of Science, CINAHL, PsycArticles and Korean database, KoreaMed and KMBASE which studied primary headache and medication-overuse headache. The primary efficacy measure was the number of headache days per month, while secondary efficacy measures were the number of headache attacks per week, headache index, treatment response rate, and migraine disability assessment. The meta-analysis was performed using R 3.5.1. to obtain pooled mean difference and pooled relative risk with 95% confidence interval (CI) for continuous data and dichotomous data, respectively.

**Results:**

From 12,773 identified articles, 27 randomized clinical trials were identified. Primary outcome showed significant superiority of psychological treatments (pooled mean difference = − 0.70, 95% CI [− 1.22, − 0.18]). For the secondary outcomes, the number of headache attacks (pooled mean difference = − 1.15, 95% CI [− 1.63, − 0.67]), the headache index (pooled mean difference = − 0.92, 95% CI [− 1.40 to − 0.44]) and the treatment response rate (pooled relative risk = 3.13, 95% CI [2.24, 4.37]) demonstrated significant improvements in the psychological treatment group over the control group.

**Conclusion:**

Psychological treatments for primary headache disorder reduced headache frequency and the headache index. Future research using standardized outcome measures and strategies for reducing bias is needed.

**Electronic supplementary material:**

The online version of this article (10.1186/s10194-019-0965-4) contains supplementary material, which is available to authorized users.

## Background

Headache disorder is very common and frequently becomes chronic. It is so common that approximately 50% of adults suffered from a headache during a 1 year period, and according to the Eurolight Project, 77% of adults in Europe experience at least one headache in their lifetime. Chronic headache, defined as headaches occurring more than 180 days a year or more than 14 days a month for more than 3 months, has also been reported to be prevalence with a 1-year prevalence rate of 4.0%, and similarly, there is a high frequency of possible medication-overuse headache with an estimated 1-year prevalence rate 1–2% [1]. Furthermore, headache significantly interferes with the daily functioning of affected patients, and according to the World Health Organization (WHO), migraine is the top leading cause of global burden of diseases in adults aged 15–49 years [2]. The estimated mean annual costs for headache per person due to direct (e.g., treatment and investigation) and indirect (e.g., work absenteeism and reduced productivity) causes were estimated at € 1222 for migraine, € 303 for tension-type headache and € 3561 for medication-overuse headache [3].

Pharmacotherapeutic approaches for the prophylaxis of headache disorder have been studied, and a wide range of medications are currently being used. Cardiovascular, antidepressant and anticonvulsant medications, such as propranolol, tricyclic antidepressants, and topiramate have demonstrated efficacy for migraine prophylaxis and are frequently used. However, the efficacy has not been entirely sufficient, and generally, no single drug has appeared to reduce headache frequency by much more than 50% in approximately half of patients [4]. This unmet need of headache prophylaxis might result from the lack of understanding the mechanism of headache. Malfunctioning in modulation of excitability of nociceptive brain circuits, cortical spreading depression for migraine and acquired central sensitization with peripheral activation for tension-type headache have been suggested as pathophysiology of headache. But there are some controversies and they cannot fully explain the pathophysiology of the headache [5–8]. Above mentioned medications seem to have effect on modulation of central sensitization by reduction of excitatory neurotransmission and facilitation of inhibitory neurotransmission but the mechanism of action are also not fully understood yet [9]. Therefore, investigation of pathophysiology of headache and novel therapeutic approach based on the pathophysiology is required. Also, more active strategies for overcome the limitation of current pharmacotherapy should be investigated. As mentioned above, medication-overuse headache is frequent in those with chronic headache disorder. Further, in certain populations, such as pregnant or lactating patients or those with allergies to certain medications, pharmacotherapy can be contraindicated, and those patients may be reluctant to use medication. For these reasons, non- pharmacotherapeutic approaches can be a good option for headache as either a monotherapy or a concomitant treatment with pharmacotherapy.

Psychological treatment can be appropriate for headache disorder. As headache disorder often become chronic and distressful, psychological factors such as stress, specific personality traits or temperament and psychiatric disorders have been reported to be frequent in headache patients. High perceived disability in patient with migraine was reported to be associated with depression and symptoms of stagnation [10]. As these comorbid conditions may negatively modify the outcome of headache disorder, such as decreased quality of life and increased suicidal risk [11, 12], psychological treatment can be help with those patients. Besides the comorbid conditions, psychological treatment might affect the headache symptom itself. For primary headache, such as migraine and tension-type headache, trigger factors have been investigated and developing strategies for managing these factors is advisable. Frequently mentioned trigger factors are menstruation, skipping meals, alcohol consumption, caffeine withdrawal, sleep problems, psychological stress and environmental factors, such as weather, light, and odors [13]. Among trigger factors, a recent meta-analysis reported that stress and sleep were most common, which are modifiable by psychological treatment [14]. Major depressive disorder and anxiety disorder also seem to increase the risk of headache and vice versa [15–18]. Some studies have identified the presence of a relationship between specific personality traits or psychological distress and headache disorder [19]. A neurolimbic model has been suggested in some headache patients in which an altered connectivity between brainstem pain-modulating circuits, including the periaqueductal gray and limbic system, exists. This model may explain the bidirectional relationship between headache and mood [20].

Cognitive behavior therapy (CBT) is frequently used and has been found to effective for stress management and sleep disorder, which is an important trigger factor of headache. Relaxation training and biofeedback (BFT) have also been widely accepted for use in treating headache. These psychological interventions have been studied and used in headache for more than four decades [21]. Evidence-based guidelines for migraine headache developed by the United States Headache Consortium recommended relaxation training, BFT, and CBT as treatment options for migraine prevention [22]. However, a more recent guideline from the National Institute for Health and Care Excellence (NICE) in the United Kingdom determined to not make a recommendation on the psychological treatment for primary headache due to the lack of empirical evidence. The guideline development group of the NICE mentioned that previous research has been poor in quality due to poor or missing control groups and small sample sizes. Thus, the potential effectiveness of psychological treatments offset the high costs of headache has been difficult to assess; however, NICE also recommended further research to strengthen evidence regarding psychological treatments [23].

The analysis of NICE guideline focused also on the cost-effectiveness and used strict inclusion criteria. They only included the studies compared psychological treatment with active controls such as pharmacological therapy, acupuncture, manual therapy, herbal remedies or dietary supplements with more than sample size of 25. As the characteristics of the most studies about psychological treatment in headache were not satisfying the inclusion criteria, especially in control group and sample size, only 5 studies included in the analysis. Psychological treatment can be preferred some specific situation as previously mentioned and might have additional benefit when used in combination manners with other treatment. So assessing the effectiveness of psychological treatment alone not compared with active control for primary headache might be beneficial. Meta-analysis of the relevant studies with well-designed protocol can overcome the shortness of sample size in single study. Further, as guideline recommendation considers the cost-effectiveness, it can be differed by countries because of different cost and accessibility. Recently in Korea, CBT has begun to be covered by national health insurance. Further, other modalities, such as mindfulness-based treatment (MBT) have evolved and are being used to treat chronic recurrent pain disorders [24, 25]. A thorough and updated review of empirical research including studies from Korea using more novel modalities would be helpful and can be the basis for recommendations regarding psychological treatment for primary headache as stand-alone treatment option or adjunctive treatment with other treatment modalities in Korea.

In this manuscript, we systematically reviewed the previous literature about psychological treatment in primary headache. Through this systematic review and meta-analysis, we tried to assess the effect of psychological treatment on headache. The main points of our meta-analysis are as follows: 1) we focused on the headache related variables as outcome result, not the effect on psychological or psychiatric conditions; 2) Try to include studies which can distinguish the effect of psychological treatment, by using studies which compared psychological treatment with treatment as usual or waiting list and also the studies compared combination of psychological treatment and other treatment modality with the other treatment modality alone, so the additive effect of psychological treatment can be assessed; 3) We tried to include the Korean manuscript to make better evidence for treatment recommendations can be used in Korea.

## Methods

### Data sources and search strategy

We searched for peer reviewed articles using the English databases of EMBASE, MEDLINE, the Cochrane Library Database, SCOPUS, Science Direct, Web of Science, CINAHL and PsycArticles. Further, the Korean database, KoreaMed, and KMBASE were searched using English and Korean search terms. We searched articles from the inception of each database to 13 March 2018. We included only studies that were written in English and Korean. We included in our search articles that were written about primary headache (e.g., migraine, tension-type headache, and cluster headache) and medication-overuse headache. Possible psychological treatments included psychotherapy, CBT, cognitive therapy, and behavior therapy, such as BFT, neurofeedback, relaxation training, autogenic training, meditation, and MBT Additional file 1. Studies that were focused on headache education, physical therapy or exercise, information dissemination, and simple counselling were excluded.

### Inclusion and exclusion criteria

We included peer-reviewed journal articles with samples of adult suffering from primary headache and randomized controlled trials (RCTs) about psychological treatment. Studies not designed as RCTs or not full publication, such as case control studies, single arm studies or conference abstracts were excluded. Studies having children or adolescent as subjects or secondary headache as target condition were also excluded. In case of articles written about multimodal interventions of non-pharmacological treatments, articles written about the combination of psychological treatments were included, but articles focused on combination of treatment such as physical therapy or acupuncture were excluded due to the difficulty with distinguishing the efficacy of those different modalities. Included studies required a control group that was “treatment as usual,” a waiting-list control, no intervention, or a pseudo-intervention. If preventive pharmacotherapy or other forms of preventive intervention were mandatory for the control group, the treatment group was required to be adjunctive psychological treatment with preventive pharmacotherapy or intervention of control group. Finally, articles were required to use headache-related efficacy measures with at least one assessment of 1) headache frequency defined as headache days or number of attacks per specific periods (e.g., a week, a month) or 2) a headache index which reflects diverse aspects of headache suffering or 3) a migraine disability assessment (MIDAS) [26]. As we used mean and standard deviations (SD) for the continuous outcome variables and percentage of responder for categorical variable for meta-analysis, studies that we could not extract the data of interest were also excluded.

### Data extraction

We extracted following information from the included RCTs: the publishing country, headache type of interest, intervention used in study arms, number of participants included in each study arm, number of participants included in the control group, duration of intervention used, outcome measures, and timing of outcome measures.

The primary efficacy measure was the headache frequency, which was defined as number of days with headache per month. The secondary efficacy measures were headache frequency defined as the number of headache attacks per week, headache index, and treatment response rate, defined as more than 50% improvement from baseline on the headache index and MIDAS following treatment.

The outcome measures about headache that were selected for use in the meta-analysis were extracted from each study. We extracted the means and SD of the outcome data at baseline and at follow-up and the number of participants in each group for continuous data. For categorical data, total number of participants, and the number of participants with treatment response were extracted from each study arms.

### Data synthesis and statistical analysis

We performed a meta-analysis using R 3.5.1 (R Core Team, 2018) [27]. For the continuous data, the pooled efficacy measure was assessed as the mean difference with a 95% confidential interval (95% CI). The differences in the outcome measures between baseline and follow-up for each study arm and control arm were calculated using the means and standard deviations at baseline and follow-up at each arm. We used the inverse variance method for the meta-analysis. For comparing the prevalence of those who responded to in both the psychological treatment group and control group, the pooled relative risk (RR) with 95% CI was calculated using the Mantel-Haenszel Method. If heterogeneity among the studies included in the identified meta-analysis was low, we applied a fixed effect model, otherwise we applied a random-effect model.

### Heterogeneity analysis, sensitivity analysis and subgroup analysis

Heterogeneity among the studies included in the meta-analysis was tested using I-squared (I^2^) statistic, with an I^2^ value that was higher than 50% being meaningful heterogeneity. Sensitivity analyses were performed by excluding one study at a time from the meta-analysis to test the robustness of the effects of a single study on the overall estimate.

We also performed a subgroup analysis to assess the influence of the following factors on the effectiveness of treatment: Headache type (restricted to migraine vs. tension-type headache vs. cluster headache vs. medication-overuse headache vs. primary headache with no restriction on headache type), type of intervention (study including CBT vs. BFT vs. MBT vs. other treatment-type without a previously mentioned treatment components), and the study location (Korea vs. United States vs. European countries vs. other countries).

### Risk of bias

We assessed the risk of bias in the studies included in the meta-analysis using a revised tool by Higgins and colleagues (2016) for assessing risk of bias in randomized trials [28]. If more than 10 studies were included in a single selected meta-analysis study, a visual inspection of the funnel plot was performed to review the reporting bias.

## Results

### Search results and quality assessment

**Fig. 1:**
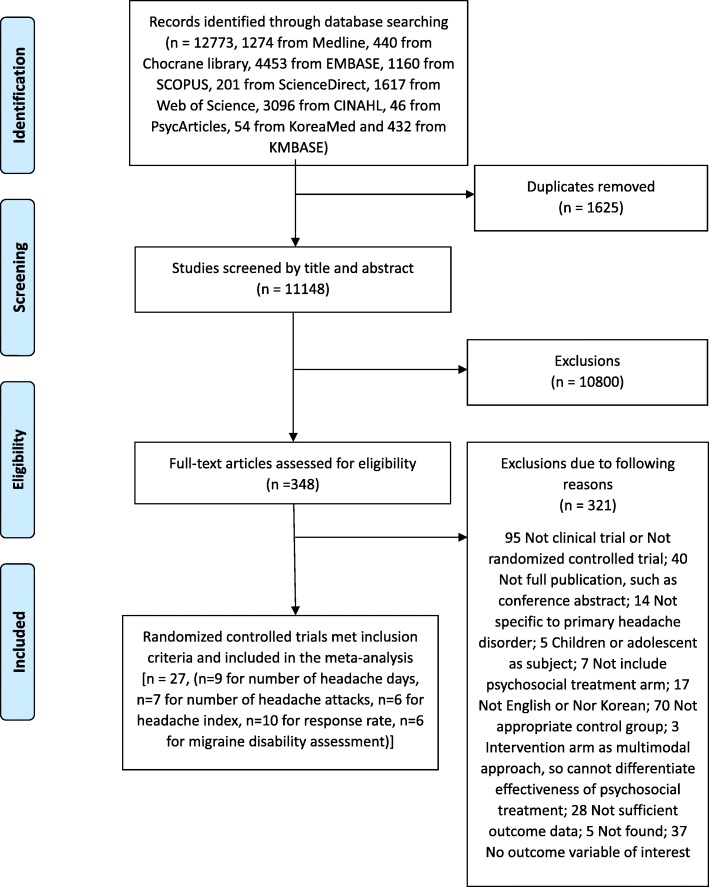
Schematic presentation of studies selected in the present meta-analysis

From the initial database search, we initially identified 12,773 articles. After the removal of articles using the same dataset and screening of the title and abstract, 348 articles remained for a full text assessment. The final sample included 27 RCTs which met our inclusion criteria. A flow diagram of our selection criteria for research articles in the meta-analysis and the reasons for exclusion is shown in Fig. 1.

The selected studies used multiple outcome measures, and nine studies were included in meta-analysis assessed the number of headache days [29–37], seven studies assessed the number of headache attacks [38–44], six studies used a headache index [45–50], 10 studies assessed the treatment response rate [35, 45, 47–54], and six studies used the MIDAS [29, 30, 32, 36, 41, 55]. The specific characteristics of the selected studies are described in Table 1. The risk of bias assessment showed that 1 study had low risk, 22 studies had some concerns and 4 studies had high risk (Table 2). As none of the identified meta-analyses included more than 10 studies, we could not examine them and rule out the risk of publication bias.

**Table 1:** Summary of characteristics of included studies

Study ID	Published country	Headache type	Intervention used in study arms (N)	Control group N	Duration of intervention	Outcome measure	Time of outcome measure
K. A. Foster et al., 2004	USA	Primary headache	Attention control (6)	12	6 weeks	Frequency attack Intensity, duration, medication usage, self-reported HQOL	6 weeks
			Trager treatment (11)				
E. B. Blanchard et al., 1991	USA	Tension type headache	Progressive muscle relaxation with home practice (14)	6	8 weeks	HI, HI improvement MI	12 weeks
			Progressive muscle relaxation without home practice (13)				
E. B. Blanchard et al., 1991	USA	Migraine	Thermal biofeedback with home practice (18)	10	6 weeks	HI MI	10 weeks
			Thermal biofeedback without home practice (19)				
T. Finn et al., 1991	USA	Tension type headache	Progressive muscle relaxation (8)	9	10 weeks	Frequency attack Duration, severity, headache free days	10 weeks, 2 months
			Rational-emotive therapy (8)				
			Headache discussion (10)				
H. Mo’tamedi et al., 2012	Iran	Primary headache	Acceptance and commitment therapy (15)	15	8 weeks	MIDAS Intensity, duration	8 weeks
D. Kewman et al., 1980	USA	Migraine	Skin temperature biofeedback,raise finger temperature (11)	11	9 weeks + 1 day	Frequency attack Duration, number of symptoms, disability, composite medication rating	15 weeks
			Skin temperature biofeedback, lower finger temperature (12)				
A. Kleiboer et al., 2014	Netherlands	Migraine	Online behavioral training in migraine self-management (195)	173	8 weeks	Frequency day, MIDAS HMSE, HSLC, MSQOL, intensity	8 weeks
P. D’Souza et al., 2008	USA	Migraine	Relaxation training (28)	31	4 weeks	Frequency day, MIDAS Severity	1 month, 3 months
			Written emotional disclosure (31)				
		Tension type headache	Relaxation training (17)	17			
			Written emotional disclosure (17)				
B. Meyer et al., 2016	Germany	Migraine	Progressive muscle relaxation (16)	19	6 weeks	Frequency day	6 weeks, 18 weeks
R. E. Wells et al., 2014	USA	Migraine	Mindfulness-based stress reduction (10)	9	8 weeks	Frequency attack, disability Severity, Duration, HIT-6, Headache management self efficacy, Migraine-specific quality of life	8 weeks, 12 weeks
V. Bembalgi et al., 2012	India	Tension type headache	Auditory galvanic skin resistance biofeedback (30)	31	Not specified (15 sessions)	Frequency attack Duration, intensity	6 months after treatment
			Visual galvanic skin resistance biofeedback (30)				
			Combined galvanic skin resistance biofeedback (30)				
O. Slavin-Spenny et al., 2013	USA	Primary headache	Relaxation training (48)	49	6 weeks	Frequency day, MIDAS Severity, duration	10 weeks
			Anger awareness and expression training (50)				
H. Mansourishad et al., 2017	Iran	Migraine	Mindfulness-based cognitive therapy (13)	13	1 month	Frequency attack Intensity, duration	1 month, 3 months
E. H. Kang et al., 2009	Korea	Migraine	Biofeedback-assisted autogenic training (17)	15	4 weeks	HI improvement	2 weeks, 4 weeks
P. Bruhn et al., 1979	Denmark	Tension type headache	Electromyographic feedback therapy (13)	10	8 weeks	HI improvement Drug intake, Duration	8 weeks, 20 weeks
K. A. Appelbaum et al., 1990	USA	Tension type headache	Progressive muscle relaxation training (16)	8	8 weeks	HI, HI improvement MI	12 weeks
			Progressive muscle relaxation training plus selected cognitive stress coping techniques (17)				
V. Bembalgi et al., 2013	India	Tension type headache	Electromyography biofeedback (23)	22	Not specified (15 sessions)	Frequency attack Intensity, duration, analgesic usage	1 month, 6 months, 12 months
			Galvanic skin resistance biofeedback (22)				
P. Martin et al., 2007	Australia	Primary headache	Cognitive behavioral therapy (18)	13	2 months	HI improvement Medication use	2 months, 6 months, 12 months
			Temporal pulse amplitude biofeedback training (19)				
L. A. Rokicki et al., 1997	USA	Tension type headache	Combined relaxation and electromyographic biofeedback therapy (30)	14	3 weeks	HI, HI improvement Headache free days, medication intake, headache activity	3 weeks
S. Cathcart et al., 2014	Australia	Tension type headache	Mindfulness-based therapy (23)	19	3 weeks	Frequency day Intensity, duration	3 weeks
J. Gauthier et al., 1983	Canada	Migraine	Temporal Artery Constriction feedback (7)	7	2 months	Frequency day Intensity, duration, medication	2 m
			Temporal artery dilation feedback (7)				
G. Pickering et al., 2012	France	Migraine	Autogenic training (29)	29	2 months	Frequency day Headache score, analgesics consumption	2 months, 4 months
T. Devineni et al., 2005	USA	Primary headache	Progressive muscle relaxation plus cognitive stress coping therapy for tension-type headache, Autogenic training plus progressive muscle relaxation for migraine or mixed headache (39)	47	1 month	HI improvement HDI, MI	1 month
S. Cousins et al., 2015	UK	Migraine	Brief guided self-help cognitive behavioral therapy and relaxation treatment (36)	37	5 weeks	Frequency day, MIDAS HIT-6, mean number of days rescue medication used	4 months
E. Blanchard et al., 1990	USA	Primary headache	Thermal biofeedback with adjunctive relaxation training (32)	54 (Pseudo-meditation 24, WL 30)	2 months	HI, HI improvement MI	2 months
			Thermal biofeedback with adjunctive relaxation training plus cognitive therapy (30)				
E. B. Blanchard et al., 1990	USA	Primary headache	Home based thermal biofeedback + relaxation training (30)	17	2 months	HI, HI improvement MI	2 months
			Home based thermal biofeedback + relaxation training plus instruction in cognitive stress coping techniques (29)				
M. Odawara et al., 2015	Japan	Migraine	Biofeedback (16)	11	10w	Frequency day Intensity, headache related disability	14 weeks

N, Number of subjects included in each study arm; HQOL, headache quality of life; HI, headache index; MI, medication index; MIDAS, migraine disability assessment; HMSE, headache management self-efficacy; HSLC, headache-specific locus of control scale; MSQOL, migraine specific quality of life; HIT-6, headache disability inventory; WL, waiting list

**Table 2:** Risk of bias of selected studies

Study ID	Randomization process	Deviations from intended interventions	Missing outcome data	Measurement of the outcome	Selection of the reported result	Overall Bias
K. A. Foster et al., 2004	Some concerns	Low	Low	Low	Low	Some concerns
E. B. Blanchard et al., 1991	Some concerns	Low	Low	Low	Some concerns	Some concerns
E. B. Blanchard et al., 1991	Some concerns	Low	Low	Low	Some concerns	Some concerns
T. Finn et al., 1991	Some concerns	Some concerns	Some concerns	Low	Some concerns	Some concerns
H. Mo’tamedi et al., 2012	Some concerns	Some concerns	High	Some concerns	Low	High
D. Kewman et al., 1980	Low	Some concerns	Some concerns	Some concerns	Low	Some concerns
A. Kleiboer et al., 2014	Low	Some concerns	High	Low	Low	High
P. D’Souza et al., 2008	Low	Low	Low	Low	Low	Low
B. Meyer et al., 2016	Some concerns	Some concerns	Some concerns	Low	Low	Some concerns
R. E. Wells et al., 2014	Low	Low	Low	Some concerns	Low	Some concerns
V. Bembalgi et al., 2012	Low	Some concerns	Some concerns	Low	Low	Some concerns
O. Slavin-Spenny et al., 2013	Low	Some concerns	Some concerns	Some concerns	Low	Some concerns
H. Mansourishad et al., 2017	Some concerns	Low	Low	Low	Low	Some concerns
E. H. Kang et al., 2009	Some concerns	Some concerns	Some concerns	Some concerns	Some concerns	Some concerns
P. Bruhn et al., 1979	Some concerns	Some concerns	High	Some concerns	Some concerns	High
K. A. Appelbaum et al., 1990	Some concerns	Some concerns	High	Some concerns	Some concerns	High
V. Bembalgi et al., 2013	Low	Some concerns	Some concerns	Low	Low	Some concerns
P. Martin et al., 2007	Some concerns	Some concerns	Low	Some concerns	Low	Some concerns
L. A. Rokicki et al., 1997	Some concerns	Some concerns	Low	Low	Some concerns	Some concerns
S. Cathcart et al., 2014	Low	Some concerns	Low	Low	Low	Some concerns
J. Gauthier et al., 1983	Some concerns	Some concerns	Some concerns	Low	Some concerns	Some concerns
G. Pickering et al., 2012	Low	Some concerns	Some concerns	Low	Low	Some concerns
T. Devineni et al., 2005	Some concerns	Some concerns	Some concerns	Some concerns	Low	Some concerns
S. Cousins et al., 2015	Low	Some concerns	Low	Low	Low	Some concerns
E. Blanchard et al., 1990	Some concerns	Some concerns	Low	Some concerns	Some concerns	Some concerns
E. B. Blanchard et al., 1990	Some concerns	Some concerns	Low	Some concerns	Low	Some concerns
M. Odawara et al., 2015	Low	Low	Some concerns	Low	Low	Some concerns

### Efficacy of psychological treatment for primary headache

#### Headache frequency measured by number of headache days

**Fig. 2:**
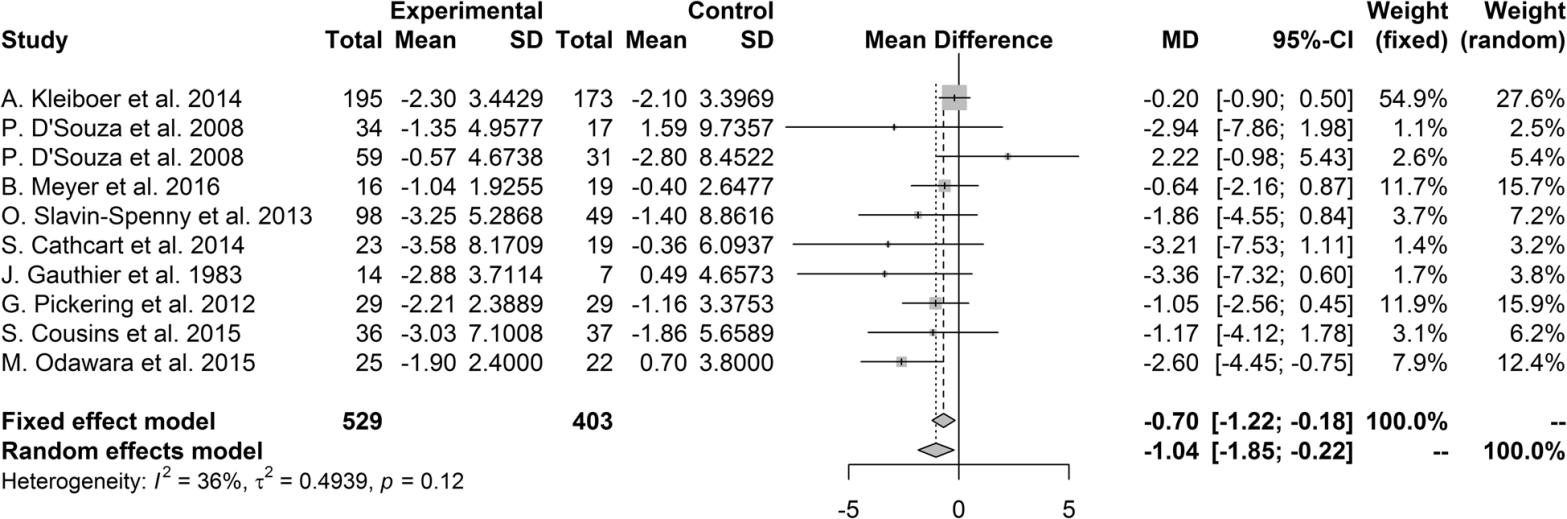
Forest plot of headache frequency measured by number of headache days

Our primary outcome measure for efficacy was days of headache per month. The pooled mean difference of any psychological treatment for any primary headache was − 0.70 (95% CI [− 1.22, − 0.18], *P* = 0.01), favoring the psychological treatment group over the control group. Our heterogeneity analysis showed that studies included in analysis were not heterogeneous (I^2^ = 36%, *P* = 0.12; Fig. 2). However, sensitivity analysis showed that when study of Odawara M et al. [37] was excluded from analysis, overall result became marginally significant with mean difference of − 0.54 (95% CI [− 1.08, 0.00], *P* = 0.05).

In subgroup analysis, there were no significant difference between subgroups when performed by headache type (*P* = 0.23) and intervention type (*P* = 0.67). According to type of headache, studies that restricted the sample to migraine significantly favored psychological treatment over placebo with pooled mean difference of − 0.59 (95% CI [− 1.12, − 0.05]). Studies that restricted the sample to tension type headache or did not restrict the sample by headache type did not show significant differences between the psychological treatment and control groups. None of the studies regarding specific intervention type showed significantly improved efficacy over control group. According to the publication country, there was significant difference between subgroups in subgroup analysis (*P* = 0.02). Studies from other countries showed significantly better results for the treatment group than the control group with mean difference of − 2.80 (95% CI [− 4.36, − 1.24]), but studies from United States and European countries did not show significant difference between groups.

#### Headache frequency measured by number of headache attacks

**Fig. 3:**
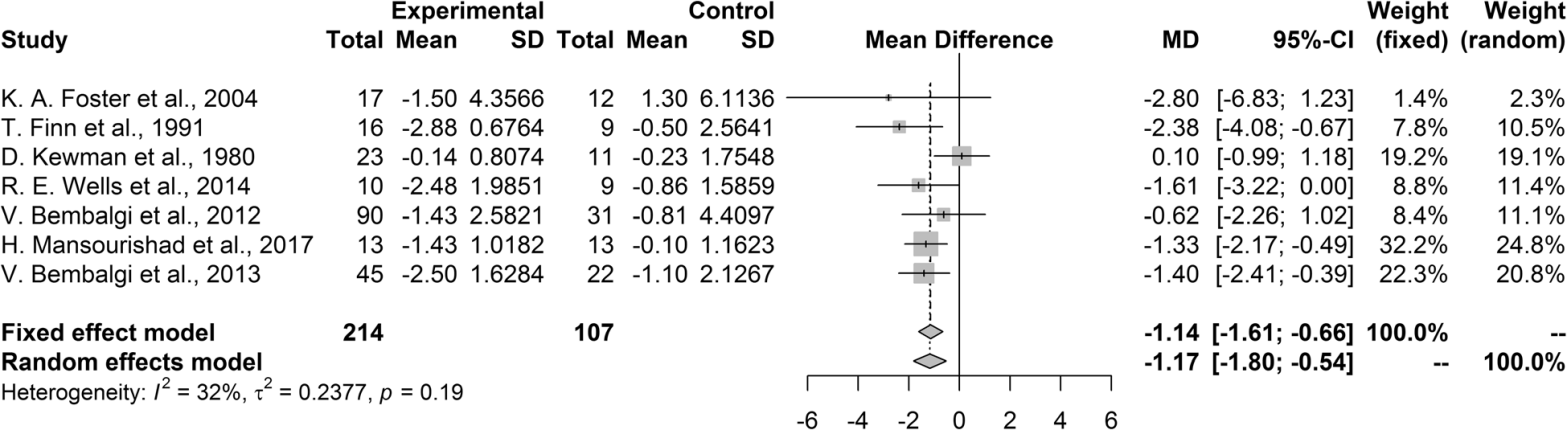
Forest plot of headache frequency measured by number of headache attacks

The pooled mean difference of the number of headaches per week was − 1.14 (95% CI [− 1.61, − 0.66, *P* < 0.001) favoring the psychological treatment group over the control group. Our heterogeneity analysis showed that studies included in analysis were not heterogeneous (I^2^ = 32%, *P* = 0.19; Fig. 3). In the sensitivity analysis, no single study robustly affected the result of meta-analysis.

In the subgroup analysis, there were no significant differences between the subgroups when analyzed by headache type (*P* = 0.55), type of intervention (*P* = 0.26) and country (*P* = 0.93). Studies that restricted the sample to migraine and tension-type headache significantly favored psychological treatment over placebo with pooled mean difference of − 0.91 (95% CI [− 1.53, − 0.30]) and − 1.43 (95% CI [− 2.19, − 0.66]), respectively. Studies that did not restrict the sample by headache type did not show significant differences between the psychological treatment and control groups. In the subgroup analysis performed by intervention type, studies using BFT or CBT or MBT showed significantly better results for the psychological treatment group than the control group with pooled mean difference of − 0.70 (95% CI[− 1.37, − 0.02], − 3.00 (95% CI [− 5.43, − 0.57]) and − 1.39 (95% CI [− 2.13, 0.64]), respectively. Studies with other treatments did not show significant differences between the psychological treatment and control groups. Subgroup analysis according to country of research revealed that studies both in United states and other countries showed significant difference between psychological treatment and control groups with pooled mean difference of − 0.94 (95% CI [− 1.72, − 0.15]) and − 1.26 (95% CI [− 1.86, − 0.66]), respectively.

#### Headache index

**Fig. 4:**
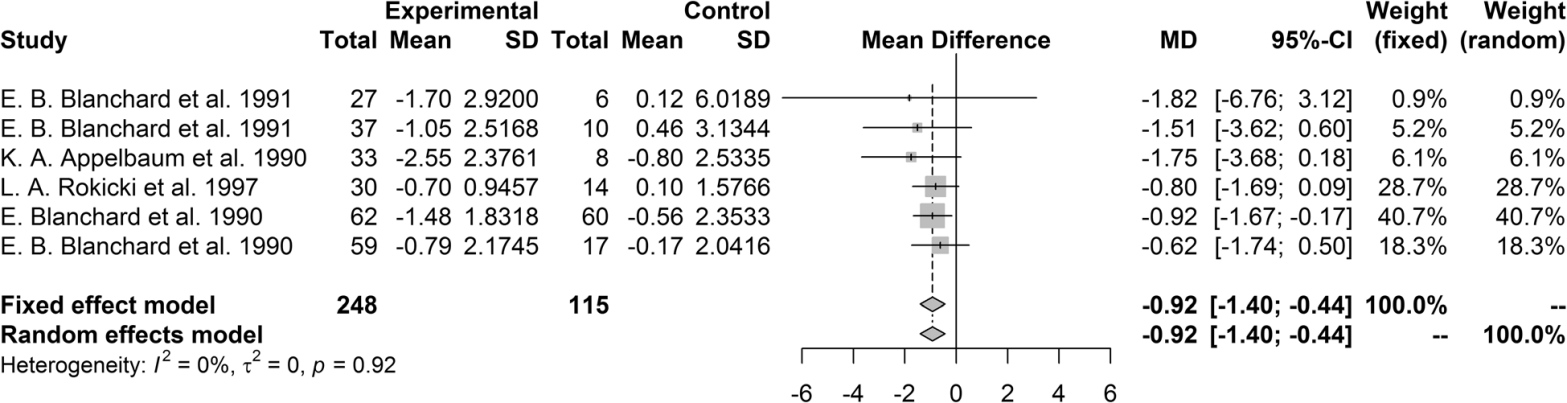
Forest plot of headache index

Headache index was analyzed in two ways: mean difference of headache index score and proportion of those who responded to treatment. The pooled mean difference of change in score of headache index score was − 0.92 (95% CI [− 1.40, − 0.44], *P* < 0.001), favoring the psychological treatment group over the control group. Our heterogeneity analysis showed that studies included in analysis were not heterogeneous (I^2^ = 0%, *P* = 0.92; Fig. 4). In the sensitivity analysis, no single study robustly affected the result of meta-analysis.

In subgroup analysis performed by headache type, there were no significant differences in results between the subgroups (*P* = 0.81). Studies that restricted the sample to those with tension-type headache and studies that did not restricted the headache type showed significantly favorable results for the psychological treatment group compared to the control group with pooled mean difference of − 0.99 (95% CI [− 1.79, − 0.19]) and − 0.83 (95% CI [− 1.45, − 0.20]), respectively. There was only one study that restricted headache type to migraine, and the study did not show significant difference between groups. In the subgroup analysis performed by intervention, there were no significant differences between subgroups (*P* = 0.83). Treatment groups using BFT showed significantly better results over the control groups with pooled mean difference of − 0.86 (95% CI[− 1.35, − 0.36]) but significant results were not found for other interventions, including CBT. There were two studies that included both BFT and CBT as a treatment, and those studies were analyzed as another, separate subgroup, and they did not show significant group differences. However, if these two studies were included with the CBT group, the results of CBT became significant (pooled mean difference = − 0.91, 95% CI [− 1.51, − 0.32]). As all of the included studies were from United States, we did not perform the subgroup analysis by country.

#### Treatment response

**Fig. 5:**
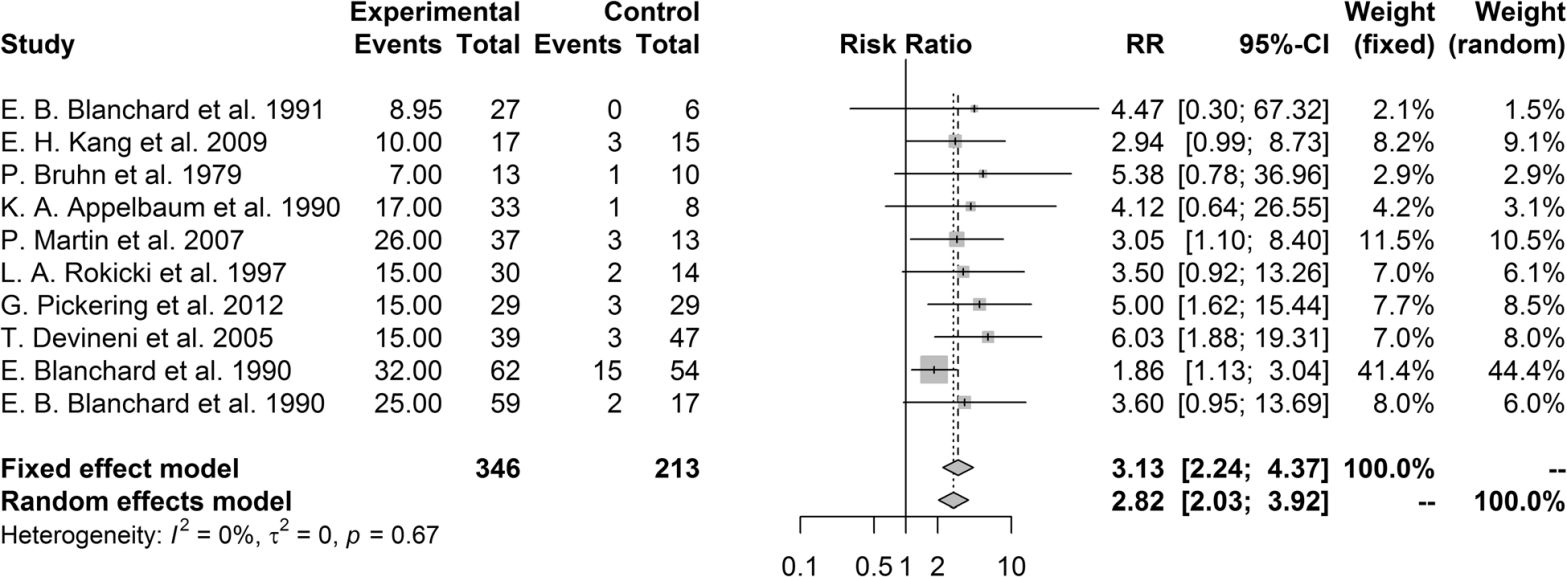
Forest plot of treatment response rate

Treatment response was more prevalent in psychological treatment group than control group with pooled RR of 3.13 (95% CI [2.24, 4.37], *P* < 0.001). There was no heterogeneity among the included studies (I^2^ = 0%, *P* = 0.67; Fig. 5). In the sensitivity analysis, no single study robustly affected the overall results.

Subgroup analysis showed that there was no significance between subgroups when divided by type of headache (*P* = 0.54), type of intervention (*P* = 0.38) and country (*P* = 0.76). According to headache type, all of the subgroups showed better results for the psychological treatment group as pooled RR of 3.94 (95% CI [1.80, 8.62]) for migraine, 4.16 (95% CI [1.70, 10.19]) for tension-type headache, and 2.70 (95% CI [1.80, 4.03]) for primary headache without restriction on headache type. All of subgroups by intervention showed a significantly higher response rate in the psychological treatment group than the control group with pooled RR of 2.74 (95% CI [1.70, 4.42]) for studies including BFT, 4.75 (95% CI [2.03, 11.12]) for studies including CBT, 4.78 (95% CI [1.79, 12.75]) for studies including other treatments, and 2.13 (95% CI [1.08, 4.21]) for studies including both BFT and CBT. In subgroup analysis by country, studies from United States (pooled RR = 2.52, 95% CI [1.70, 3.74]), European countries (pooled RR = 5.10, 95% CI [1.93, 13.48]) and other countries (pooled RR = 3.05, 95% CI [1.10, 8.40]) showed significantly higher response rate in the psychological treatment group than the control group. There was only one study from Korea and it could not show significant result.

#### Disability due to headache

**Fig. 6:**
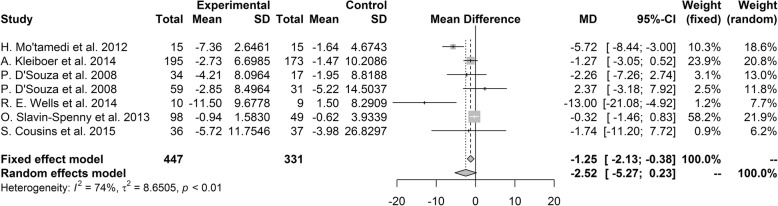
Forest plot of migraine disability assessment

Studies which measured disability due to headache by MIDAS were included in the meta-analysis. The pooled mean difference of MIDAS was − 2.52 (95% CI [− 5.27, 0.23], *P* = 0.073), suggesting a favorable trend for the psychological treatment group over control group, but this difference was not statistically significant. Our heterogeneity analysis showed that studies included in analysis were significantly heterogeneous (I^2^ = 74%, *P* < 0.01; Fig. 6). In the sensitivity analysis, the migraine group from a study by D’Souza et al. [22] robustly affected the overall results, and the results became significant when the studies arm was excluded with mean difference of − 3.15 (95% CI [− 6.04, − 0.27], *P* = 0.03).

In subgroup analysis, there were no significant difference between subgroups divided by headache type (*P* = 0.98) and type of intervention (*P* = 0.05). No subgroup divided by headache type showed that treatment group was significantly favorable over control group. Subgroup analysis by type of intervention revealed significant better result in studies using MBT over control group (mean difference = − 13.00, 95% CI [− 21.08, − 4.92]), but subgroups using BFT, CBT and other interventions did not show significant differences between treatment groups and control groups. According to the country of research, there was significant difference between subgroups (*P* = 0.03). Studies from other countries showed significantly better results for the treatment group than the control group with mean difference of − 5.72 (95% CI [− 8.44, − 3.0]), but studies from United States and European countries did not show significant difference between groups.

## Discussion

In this meta-analysis, we found that psychological treatments for primary headache disorder are effective for headache itself not only for addressing the concomitant psychological distress. Pooled results for the frequency of headache and headache index were found to demonstrate significant improvements for treatment groups compared to the control groups. Although headache-related disability was not significantly influenced by psychological treatment, the results indicated better outcomes for the treatment group compared to the control group. Further, when excluding the results of a migraine group study by D’Souza et al., the pooled efficacy on headache-related disability became significant [30]. In that article we included both the relaxation treatment group and the written emotional treatment group as a combined psychological treatment group, although the article showed that relaxation training had better treatment outcome than the control group while the written emotional treatment group showed no improvement. This incorporation of intervention without efficacy might reduce the overall effect size of our meta-analysis. In the case of the migraine group in the study, there were no significant differences between groups in terms of frequency and disability. The authors concluded that lack of efficacy for migraine was due to the brevity of the intervention, as migraine treatment is more challenging and may require more comprehensive relaxation training, which was supported by their finding that relaxation training was effective for pain severity for migraines [30].

During our database search, we identified some articles which compared directly the effectiveness of pharmacotherapy and psychological therapy for the treatment of primary headache. While these studies were not included in the meta-analysis, as they did not meet the criteria of having a control group, psychological therapy (e.g., CBT or BFT with relaxation) showed comparable effects to pharmacotherapy (e.g., amitriptyline or propranolol) [56, 57]. Further, the combination of pharmacotherapy (e.g., amitriptyline or propranolol) with psychological therapy (e.g., biofeedback) was found to be more effective than pharmacotherapy or psychological therapy alone [58]. Other studies with multidisciplinary programs, including psychological intervention combined with physical therapy or pharmacotherapy, were excluded in this meta-analysis due to challenges with confirming the individual influence of psychological treatment. These studies also showed the effectiveness of multimodal non-pharmacotherapeutic approach using psychological intervention for primary headache [59–61]. As the pathophysiology of headache is not fully understood, the mechanism of psychological treatment in headache is somewhat unclear. Management and regulation of major trigger factors of headache, such as stress, emotional experience and sleep or comorbid psychopathology which interact with headache bidirectionally by psychological treatment might exert preventive effect on headache. Further, physiological changes from psychological treatment, such as modulation of endogenous opioids system, change in sympathetic activity, or modulation of pain-related brain neuroplasticity may also affect headache and pain [62–64].

Although our results are promising, there are some limitations to their interpretation. The diversity of treatment modality and the heterogeneity of the specific protocols for each modality could influence the outcome variables. We performed subgroup analyses by categorizing the types of treatment. A number of studies used a combination of treatment modalities, such as CBT along with relaxation training. As it was difficult to assess the individual efficacy of very specific treatment modalities, we opted to select some treatment modalities of interest (i.e., BFT, CBT and MBT) and then divided the studies by their inclusion of such interventions as a part of treatment. Although we covered all treatments combined with BFT as being the same, specific studies included in the meta-analysis had different protocols, such as hand warming, hand cooling, galvanic skin resistance, electromyography, temporal artery constriction, and temporal artery dilation. Furthermore, some studies directly compared BFT using different protocols and showed that some protocols were more effective than other [42, 44]. In those studies, we included both BFT protocols as the treatment group without discriminating between the protocols. In the case of MBT, there were also numerous types of treatments, such as mindfulness-based cognitive therapy, mindfulness-based stress reduction, and mindfulness meditation [65–69]; however, we covered all treatments using mindfulness as a single type of treatment for the meta-analysis. This could lead to heterogeneity and influence the effect size. The lack of standardized treatment protocols in psychological treatment for primary headache a large challenge for understanding the effectiveness of treatments and making recommendations.

A large number of publications were detected through our initial search but only a small number of studies were included in the final meta-analysis. This small number of included studies might contribute to the insignificant findings for disability, as well as the results from the subgroup analysis. Present study could not find significance in many subgroup-analyses due to lack of statistical power from small number of included studies, despite of significant effectiveness in overall assessment with no significant between-group heterogeneity. One reason was that many clinical trials were not designed as RCTs. Another reason was that the identified studies used heterogeneous outcome measures, which made it difficult to extract the necessary data, leading to many RCTs being dropped from the analysis. Sufficient number of well-designed RCTs would help us to assess the efficacy of specific psychological treatment for the specific type of headache and to make more specified and tailored recommendations. Thus, we would recommend the use of more standardized outcome measures in RCTs to allow for the comparison across studies, such as through meta-analyses.

Most of the information about headache itself was obtained by self-monitoring using headache diaries and typically the collection of some of following variables: frequency, intensity or severity, duration, associated symptoms, and related medication consumption [23, 70]. Many studies used headache diary but a substantial portion of them did not give detailed information about the diary and its procedures for us. Some gave reference of the diary which was too diverse [71–74]. Among specific headache-related outcomes, intensity and duration are difficult to standardize across users and may contain some uncertainty [75]. For this reason, headache frequency is recommended as an outcome variable for RCTs of headaches [23, 75, 76]. Headache frequency can be defined by frequency of attacks or the number of days with headache. However, counting individual headaches can be challenging due to problems distinguishing between separate attacks and recurrences. Thus, the number of days with headache can be a simpler alternative [75]. In this review, we used the number of days with headache as a primary outcome but also used the number of headache attacks as a secondary outcome measure. Our result found that both of outcome measures could show the clinical efficacy that those variables can be used as an alternative measure for the other. But the significance of the pooled effect was larger in number of attacks than headache days, implying that number of attacks can be more sensitive to detect the treatment efficacy.

For reflecting on the overall suffering of patients, headache index which considering intensity or duration along with frequency was also widely used. But like headache diary there was also no reference or uniformed definition for the headache index, except a definition from Blanchard and Andrasik [71]. Despite this limitation, it has been suggested that clinically significant reductions in headache index can be more appropriate measure for recurrent migraine or chronic tension-type headache [77]. In this meta-analysis, the proportion with more than a 50% improvement in the headache index from baseline in all definition and the pooled mean difference of headache index which was defined by Blanchard and Andrasik were used as secondary outcome variable. Both results showed good discrimination between the treatment and control group with minimal heterogeneity among included studies.

The primary reason that the NICE guidelines did not make recommendation about psychological treatment for headache was due to the poor quality of available research. Our risk of bias assessment also showed that most of the included studies had some concerns or a high risk of bias. Some specific characteristics of the psychological treatments affected the risk of bias. First, it is hard to blind the group assignment in psychological treatment, so participants are more likely to know what their group assignment, and the primary headache-related outcomes are typically self-reported headache diary. This factor increased the risk of bias due to deviations from the intended intervention and the measurement of outcome variables. Some studies reduced this bias by using pseudo-treatment groups, assessing the results with an intention-to-treat analysis and including more objective outcomes, such as headache days.

Second, the relatively high rate of attrition increased the risk for bias in missing outcome data. The reasons for dropout were various, including loss of interest and having problems with making a regular appointment. Psychological treatments are more time consuming than taking medication and require the active participation and motivation of participants, thus patients without enough time or effort might have problem maintaining the treatment. Some of the included studies were rated as low risk in this regard, and those studies used the intention-to-treat method with last observation carried forward for dealing missing outcome data and/or gave information about the proportion and reason for attrition in each group and the impact of missing data.

Although the risk of bias is present, the evidence of effectiveness in using psychological treatment for headache cannot be ignored. Further, pharmacotherapy has been shown to have limited efficacy, and some population have difficulty in taking medicine. In such case, psychological treatment can be effectively used with or without medication. The possible high cost and considerable effort is a major obstacle for psychological treatments. The NICE guidelines mentioned that “In the absence of good evidence on the effectiveness of psychological therapies, it is difficult to judge whether their costs would be offset by their effectiveness at reducing headache frequency” [23]. The guideline from one country should be evaluated for its acceptability when considering its use in another country. The problem of the treatment costs largely differs among countries. In Korea, the fees for biofeedback or mindfulness-based treatment are not covered by the national health insurance, and the costs vary depending on the clinic. The costs for CBT by psychiatrist or neurologist is about $40 for an individual and about $11 for a group therapy session, and the fee is covered by insurance, although primary headache is not an indication yet made by the national health insurance [78, 79]. Further, the treatment duration of the included studies was generally about 1–2 months. As most of the psychological treatments provide a self-help technique, the treatment efficacy might be long-lasting. One study found that 5 years after completing biofeedback and/or relaxation, about 91% of migraine patients and 78% of tension-type headache patients continued to show significant improvement [80]. In direct comparison with prophylaxis using propranolol, biofeedback and relaxation have shown a similar treatment response immediately after treatment and significantly better response one-year post-treatment than did those using propranolol for migraine [57].

## Conclusions

Psychological treatment reduced headache frequency and the suffering from headaches as measured by headache index. Psychological treatment can be considered as possible option for the management of primary headache as stand-alone treatment in some specific situations and as in combination treatment in treatment resistant patients. For building more concrete evidence and making clearer recommendation, future research should use standardized outcome measures and strategies to reduce bias, such as pseudo-treatment for control groups. Further effort to build a standardized protocol or manual of psychological treatment for primary headache would be beneficial.

## Additional file

Additional file 1: **Table S1.** Database search strategy. (DOCX 30 kb)
