# Supplementary material for: Efficacy of psychological treatment for headache disorder: a systematic review and meta-analysis
Source: J Headache Pain. 2019 Feb 14;20(1):17. doi: 10.1186/s10194-019-0965-4 (PMC6734438; doi:10.1186/s10194-019-0965-4)
Supplement: Supplementary file 1 — Table S1. Database search strategy. (DOCX 30 kb) [file 10194_2019_965_MOESM1_ESM.docx]

**Supplemental table 1. Database search strategy**

**Medline search strategies**

| 1. HEADACHE/  2. exp Headache Disorders/  3. (headache* or migraine*).ti,ab.  4. exp HEADACHE DISORDERS, PRIMARY/ or HEADACHE DISORDERS/  5. exp Migraine Disorders/  6. migraine*.ti,ab.  7. Tension-Type Headache/  8. (headache* adj3 (tension or tension type or muscle contraction or psychomyogenic or stress or ordinary or essential or idiopathic or psychogenic)).tw.  9. ((chronic adj2 daily adj2 headache*) or (daily adj2 persistent adj2 headache*)).ti,ab.  10. CLUSTER HEADACHE/  11. (cluster adj4 headache*).tw.  12. ((ciliary or migrain* or petrosal or sluder* or spheno-palatine or vidian) adj4 neuralgi*).tw.  13. ((rebound or transformed) adj5 (headache* or migrain*)).ti,ab.  14. ((medication or drug or pain?killer* or ergot* or analges* or triptan* or apioid or caffeine) adj5 (over?use or mis?use or associated or induced or abuse) adj5 (headache* or migrain*)).ti,ab.  15. 1 or 2 or 3 or 4 or 5 or 6 or 7 or 8 or 9 or 10 or 11 or 12 or 13 or 14  16. LETTER/  17. EDITORIAL/  18. exp Historical Article/  19. Anecdotes as Topic/  20. COMMENT/  21. Case Reports/  22. animals/ not humans/  23. exp Animals, Laboratory/  24. exp Animal Experimentation/  25. exp Models, Animal/  26. exp RODENTIA/  27. 16 or 17 or 18 or 19 or 20 or 21 or 22 or 23 or 24 or 25 or 26  28. 15 not 27  29. Cognitive Therapy/  30. exp Biofeedback, Psychology/ or feedback/ or feedback, psychological/ or autogenic training/  31. Breathing Exercises/  32. Relaxation Therapy/  33. Muscle Relaxation/  34. "Imagery (Psychotherapy)"/  35. MEDITATION/  36. Mind-Body Therapies/ or Mind-Body Relations, metaphysical/  37. PSYCHOTHERAPY/  38. (cognitive adj behavio?r adj (therap* or treatment or technique*)).ti,ab.  39. (neurofeedback or biofeedback).ti,ab.  40. ((controlled or paced or therap* or exercise*) adj2 breathing).ti,ab.  41. (respirat* adj3 (training or exercise* or therap*)).ti,ab.  42. (CBT or qigong).ti,ab.  43. (guided adj2 (imagery or visuali*)).ti,ab.  44. (mindfulness or meditation or attention* control training).ti,ab.  45. ((finger or hand) adj2 warming).ti,ab.  46. (handwarming or fingerwarming).ti,ab.  47. (relaxation adj2 (therap* or training)).ti,ab.  48. (relaxation adj2 (muscle* or progressive or therap* or exercis* or technique* or training)).ti,ab.  49. 29 or 30 or 31 or 32 or 33 or 34 or 35 or 36 or 37 or 38 or 39 or 40 or 41 or 42 or 43 or 44 or 45 or 46 or 47 or 48  50. 28 and 49 |
| --- |

**Cochrane search strategies**

| #1 MeSH descriptor: [Headache] explode all trees  #2 MeSH descriptor: [Headache Disorders] explode all trees  #3 (migraine* or headache*):ti,ab  #4 MeSH descriptor: [Migraine Disorders] explode all trees  #5 migraine*:ti,ab  #6 MeSH descriptor: [Tension-Type Headache] this term only  #7 (headache near/3 (tension or "tension type" or "muscle contraction" or idiopathic or ordinary or psychogenic or psychomyogenic or daily or essential)):ti,ab  #8 MeSH descriptor: [Cluster Headache] this term only  #9 (cluster near/4 headache*):ti,ab  #10 ((ciliary or migrain* or petrosal or sluder* or spheno-palatine or vidian) near/4 neuralgi*):ti,ab  #11 ((Harris-Horton* or horton) near/2 (disease or headache* or syndrome*)):ti,ab  #12 ((rebound or transformed) near/5 (headache* or migrain*)):ti,ab  #13 ((medication or drug or painkiller* or pain-killer* or (pain killer*) or ergot* or analges* or triptan* or opioid or caffeine) near/5 (overuse or over-use or misuse or mis-use or associated or induced or abuse) near/5 (headache* or migrain*)):ti,ab  #14 {or #1-#13}  #15 MeSH descriptor: [Historical Article] explode all trees  #16 MeSH descriptor: [Anecdotes as Topic] this term only  #17 MeSH descriptor: [Case Reports] this term only  #18 MeSH descriptor: [Animals] this term only  #19 MeSH descriptor: [Humans] this term only  #20 MeSH descriptor: [Animals, Laboratory] explode all trees  #21 MeSH descriptor: [Animal Experimentation] explode all trees  #22 MeSH descriptor: [Models, Animal] explode all trees  #23 MeSH descriptor: [Rodentia] explode all trees  #24 {or #15-#18, #20-#23} not #19  #25 #14 not #24  #26 MeSH descriptor: [Psychotherapy] this term only  #27 MeSH descriptor: [Cognitive Therapy] this term only  #28 MeSH descriptor: [Feedback, Psychological] explode all trees  #29 MeSH descriptor: [Feedback] this term only  #30 MeSH descriptor: [Autogenic Training] this term only  #31 MeSH descriptor: [Breathing Exercises] this term only  #32 MeSH descriptor: [Relaxation] explode all trees  #33 MeSH descriptor: [Relaxation Therapy] explode all trees  #34 MeSH descriptor: [Muscle Relaxation] this term only  #35 MeSH descriptor: [Imagery (Psychotherapy)] this term only  #36 MeSH descriptor: [Mind-Body Therapies] this term only  #37 MeSH descriptor: [Mind-Body Relations, Metaphysical] this term only  #38 (cognitive next (behaviour* or behavior* or therap* or technique*)):ti,ab  #39 (neurofeedback or biofeedback or CBT or qigong or handwarming or fingerwarming or hadn-warming or finger-warming):ti,ab  #40 ((controlled or paced or therap* or exercis*) near/3 breathing):ti,ab  #41 (respirat* next (training or exercis* or therap*)):ti,ab  #42 (guided next (imagery or visuali*)):ti,ab  #43 (mindfulness or meditation or "attention* control training"):ti,ab  #44 ((finger or hand) next warming):ti,ab  #45 (relaxation near/2 (muscle* or progressive or therap* or exercis* or technique* or training)):ti,ab  #46 {or #26-#45}  #47 #25 and #46 |
| --- |

**EMBASE search strategies**

| - #51 #49 AND #50 - #50 #24 OR #25 OR #26 OR #27 OR #28 OR #29 OR #30 OR #31 OR #32 OR #33 OR #34 OR #35 OR #36 OR #37 OR #38 OR #39 OR #40 OR #41OR #42 OR #43 OR #44 OR #45 OR #46 - #49 #47 NOT #48 - #48 #13 OR #14 OR #15 OR #16 OR #17 OR #18 OR #19 OR #20 OR #21 OR #22 OR #23 - #47 #1 OR #2 OR #3 OR #4 OR #5 OR #6 OR #7 OR #8 OR #9 OR #10 OR #11 OR #12 - #46 (relaxation NEAR/2 (muscle* OR progressive OR therap* OR exercis* OR technique* OR training)):ab,ti - #45 'finger warming':ab,ti OR fingerwarming:ab,ti OR 'hand warming':ab,ti OR handwarming:ab,ti - #44 mindfulness:ab,ti OR meditation:ab,ti OR 'attention* control training':ab,ti - #43 (mindfulness:ti,ab OR meditation:ti,ab OR attention*:ti,ab) AND control:ti,ab AND training:ti,ab - #42 (guided NEAR/2 (imagery OR visuali*)):ab,ti - #41 qigong:ab,ti - #40 (respirat* NEAR/3 (training OR exercise* OR therap*)):ab,ti - #39 ((controlled OR paced OR exercise* OR therap*) NEAR/2 breathing):ab,ti - #38 neurofeedback:ab,ti OR biofeedback:ab,ti - #37 cbt:ab,ti - #36 cognitive:ti,ab AND behavio?r*:ti,ab AND near:ti,ab AND (therap*:ti,ab OR treatment:ti,ab OR technique*:ti,ab) - #35 'warming'/de - #34 'psychotherapy'/de - #33 'meditation'/de - #32 'guided imagery'/de - #31 'smooth muscle relaxation'/de OR 'muscle relaxation'/de - #30 'relaxation training'/de - #29 'breathing exercise'/de - #28 'neurofeedback'/de OR 'neurofeedback training'/de - #27 'feedback system'/de OR 'autogenic training'/de - #26 feedback AND system - #25 'psychophysiology'/de - #24 'cognitive therapy'/de - #23 'rodent'/exp - #22 'animal models'/exp - #21 'animal experiment'/exp - #20 'experimental animal'/exp - #19 'experimental animal'/de - #18 'nonhuman'/de - #17 'animal'/de NOT 'human'/de - #16 'case report'/de OR 'case study'/de - #15 editorial.pt. - #14 note.pt. - #13 'letter' OR letter.pt. - #12 ((medication OR drug OR pain?killer* OR argot* OR analges* OR triptan* OR apioid OR caffeine) NEAR/5 (over?use OR mis?use OR associated OR induced OR abuse) NEAR/5 (headache* OR migrain*)):ab,ti - #11 ((rebound OR transformed) NEAR/5 (headache* OR migrain*)):ab,ti - #10 (ciliary OR migrain* OR petrosal OR sluder* OR 'spheno palatine' OR vidian) NEAR/4 neuralgi* - #9 cluster NEAR/4 headache* - #8 'cluster headache'/exp - #7 ((chronic NEAR/2 daily NEAR/2 headache*):ab,ti) OR ((daily NEAR/2 persistent NEAR/2 headache*):ab,ti) - #6 headache* NEAR/3 (tension OR 'tension type' OR 'muscle contraction' OR psychomyogenic OR stress OR ordinary OR essential OR idiopathic OR psychogenic) - #5 'tension headache'/exp - #4 migraine*:ab,ti - #3 'migraine'/exp - #2 headache*:ab,ti OR migraine*:ab,ti - #1 'headache'/de OR 'migraine'/de OR 'primary headache'/de OR 'chronic daily headache'/de OR 'migraine aura'/de OR 'migraine with aura'/de OR 'migraine without aura'/de |
| --- |

**SCOPUS search strategies**

| TITLE-ABS ( headache* AND ( "Psychosocial treatment" OR psychotherapy OR "cognitive behavior treatment" OR cbt OR "cognitive therapy" OR "behavior therapy" OR mindfulness OR biofeedback OR neurofeedback OR "relaxation training" OR "autogenic training" OR meditation OR "mindfulness based treatmement" ) ) |
| --- |

**ScienceDirect search strategies**

| Title, abstract, keywords: Headache* AND ("Psychosocial treatment" OR psychotherapy OR "cognitive behavior treatment" OR CBT OR "cognitive therapy" OR "behavior therapy" OR mindfulness OR biofeedback OR neurofeedback OR "relaxation training" OR "autogenic training" OR meditation OR "mindfulness based treatmement") |
| --- |

**Web of Science search strategies**

| #38 #19 AND #37  DocType=All document types; Language=All languages;  #37 #36 OR #35 OR #34 OR #33 OR #32 OR #31 OR #30 OR #29 OR #28 OR #27 OR #26 OR #25 OR #24 OR #23 OR #22 OR #21 OR #20  DocType=All document types; Language=All languages;  #36 TS=( relaxation AND ( muscle* OR progressive OR therap* OR exercis* OR technique* OR training ) )  DocType=All document types; Language=All languages;  #35 TS=( relaxation AND ( therap* OR training ) )  DocType=All document types; Language=All languages;  #34 TS=( ( finger OR hand ) AND warming ) OR TS=( handwarming OR fingerwarming )  DocType=All document types; Language=All languages;  #33 TS=( mindfulness OR meditation OR "attention* control training" )  DocType=All document types; Language=All languages;  #32 TS=( guided AND ( imagery OR visuali* ) )  DocType=All document types; Language=All languages;  #31 TS=( cbt OR qigong )  DocType=All document types; Language=All languages;  #30 TS=( respirat* AND ( training OR exercise* OR therap* ) )  DocType=All document types; Language=All languages;  #29 TS=(( controlled OR paced OR therap* OR exercise* ) AND breathing )  DocType=All document types; Language=All languages;  #28 TS=( neurofeedback OR neurofeedback )  DocType=All document types; Language=All languages;  #27 TS=( cognitive AND behavio?r AND ( therap* OR treatment OR technique* ) )  DocType=All document types; Language=All languages;  #26 TS=( psychotherapy OR warming )  DocType=All document types; Language=All languages;  #25 TS=( meditation OR "Mind-Body Therapies" OR "Mind-Body Relations, metaphysical" )  DocType=All document types; Language=All languages;  #24 TS=( "Imagery (Psychotherapy)" OR "guided imagery" )  DocType=All document types; Language=All languages;  #23 TS=( "relaxation therapy" OR "Muscle Relaxation" OR "Relaxation" OR "relaxation training" OR "smooth muscle relaxation" OR "muscle relaxation" )  DocType=All document types; Language=All languages;  #22 TS=( "Breathing Exercises" OR "breathing exercise" )  DocType=All document types; Language=All languages;  #21 TS= ( "Biofeedback, Psychology" OR "feedback" OR "feedback, psychological" OR "autogenic training" OR "psychophysiology" OR "feedback system" )  DocType=All document types; Language=All languages;  #20 TS=("Cognitive Therapy" )  DocType=All document types; Language=All languages;  #19 #13 NOT #18  DocType=All document types; Language=All languages;  #18 #17 OR #16 OR #15 OR #14  DocType=All document types; Language=All languages;  #17 TS=(Rodentia or note or "case study" or nonhuman or "experimental animal" or "animal experiment" or "animal model" or "rodent")  DocType=All document types; Language=All languages;  #16 TS=("Animals, Laboratory" or "Animals Experimentation" or "Models, Animal" )  DocType=All document types; Language=All languages;  #15 TS=(animals) NOT TS=(humans)  DocType=All document types; Language=All languages;  #14 TS=(letter or editorial or "historical article" or "anecdotes as Topic" or comment or "case report")  DocType=All document types; Language=All languages;  #13 #12 OR #11 OR #10 OR #9 OR #8 OR #7 OR #6 OR #5 OR #4 OR #3 OR #2 OR #1  DocType=All document types; Language=All languages;  #12 TS=( ( medication OR drug OR pain?killer* OR ergot* OR analges* OR triptan* OR opioid OR caffeine ) AND ( over?use OR mis?use OR associated OR induced OR abuse ) AND ( headache* OR migrain* ) )  DocType=All document types; Language=All languages;  #11 TS=( ( rebound OR transformed ) AND ( headache* OR migraine* ) )  DocType=All document types; Language=All languages;  #10 TS=((ciliary or migraine* or petrosal or sluder* or "spheno-palatine" or vidian) and neuralgi*)  DocType=All document types; Language=All languages;  #9 TS=(cluster and headache*)  DocType=All document types; Language=All languages;  #8 TS=("Tension-Type Headache" or "tension headache" or "cluster headache")  DocType=All document types; Language=All languages;  #7 TS=("migraine disorders" or "migraine")  DocType=All document types; Language=All languages;  #6 TS=("migraine aura" or "migraine with aura" or "migraine without aura")  DocType=All document types; Language=All languages;  #5 TS=("headache" or "migraine" or "primary headache" or "chronic daily headache" or "migraine")  DocType=All document types; Language=All languages;  #4 TS=(headache* or migraine*) or TS=("headache disorders, primary")  DocType=All document types; Language=All languages;  #3 TS=("headache disorders")  DocType=All document types; Language=All languages;  #2 TS=("headache and facial pain")  DocType=All document types; Language=All languages;  #1  TS=(headache)  DocType=All document types; Language=All languages; |
| --- |

**CINAHL search strategies**

| S14 S12 AND S13  S13 S7 OR S8 OR S9 OR S10  S12 S11 not S6  S11 S1 OR S2 OR S3 OR S4 OR S5  S10 TX ( controlled n2 breathing or paced n2 breathing or breathing n2 therap* or breathing n2 exercise* or respirat* n3 training or respirat* n3 exercise* or respirat* n3 therap* ) OR TX ( finger n2 warming or hand n2 warming or handwarming or fingerwarming or hand-warming or finger-warming ) OR TX ( relaxation n2 therap* or relaxation n2 training or progressive n3 relaxation or relaxation n3 exercise* or relaxation n3 technique* )  S9 TX ( cognitive n3 therap* or cognitive n2 behavior or cognitive n3 technique* or cognitive n2 behavior ) OR TX ( neurofeedback or biofeedback or CBT or qigong or guided n2 imagery or visualization or guided n2 visuali* ) OR TX ( mindfulness or meditation or attention* control training )  S8 MH mind body techniques OR MH meditation OR MH qigong  S7 MH biofeedback OR MH cognitive therapy OR MH autogenic training OR MW breathing exercises+ OR MH simple relaxation therapy OR MH relaxation therapy OR MH progressive muscle relaxation OR MH relaxation techniques OR MH muscle relaxation OR MH guided imagery OR MH simple guided imagery OR MH psychotherapy  S6 PT letters OR PT editorial OR PT notes OR MH letters OR MH case report OR MH case study OR MH ( animals not human ) OR MH nonhuman OR MH experimental animals OR MH animal experimentation OR MH animal model OR MH rodents  S5 TX horton N2 headache* OR TX horton N2 syndrome* OR MH Migraine OR TX Migraine* OR TX menstrau* OR TI ( (rebound or transformed) n5 (headache* or migrain*) ) OR AB ( (rebound or transformed) n5 (headache* or migrain*) ) OR TI ( (medication or drug or painkiller* or pain-killer* or pain killer* or ergot* or analges* or triptan or opioid or caffeine) n5 (overuse or over-use or misuse or mis-use or associated or induced or abuse) n5 (headache* or migraine*) ) OR AB ( (medication or drug or painkiller* or pain-killer* or pain killer* or ergot* or analges* or triptan or opioid or caffeine) n5 (overuse or over-use or misuse or mis-use or associated or induced or abuse) n5 (headache* or migraine*) )  S4 MH cluster headache OR TX cluster n4 headache* OR TX ciliary n4 neuralgi* OR TX migrain* n4 neuralgi* OR TX petrosal n4 neuralgi* OR TX sluder* n4 neuralgi* OR TX spheno-palatine n4 neuralgi* OR TX vidian n4 neuralgi* OR TX Harris-Horton* N2 disease OR TX Harris-Horton* N2 headache* OR TX Harris-Horton* N2 syndrome* OR TX horton N2 disease    S3 MH tension headache OR TX headache* n3 tension* OR TX headache* n3 "muscle contraction" OR TX headache* n3 psychomyogenic OR TX headache* n3 stress OR TX headache* n3 ordinary OR TX headache* n3 essential OR TX headache* n3 idiopathic OR TX headache* n3 psychogenic OR TX headache* n3 daily    S2 MH headache disorders OR MH migraine OR MH primary headaches OR MH chronic daily headache OR MH migraine with aura OR MH migraine without aura    S1 MH headache+ OR TI ( headache* or migraine* ) OR AB ( headache* or migraine* ) |
| --- |

**PsycArticles Search strategies**

| #1 MJMAINSUBJECT.EXACT.EXPLODE("Headache")  #2 ti(Headache*) OR ab(Headache*)  #3 MJMAINSUBJECT.EXACT.EXPLODE("Cognitive Behavior Therapy")  #4 MJMAINSUBJECT.EXACT.EXPLODE("Cognitive Therapy")  #5 MJMAINSUBJECT.EXACT.EXPLODE("Behavior Therapy")  #6 MJMAINSUBJECT.EXACT.EXPLODE("Mindfulness")  #7 MJMAINSUBJECT.EXACT.EXPLODE("Biofeedback")  #8 MJMAINSUBJECT.EXACT.EXPLODE("Relaxation Therapy")  #9 MJMAINSUBJECT.EXACT.EXPLODE("Autogenic Training")  #10 MJMAINSUBJECT.EXACT.EXPLODE("Meditation")  #11 ti("Psychosocial treatment" OR psychotherapy OR "cognitive behavior treatment" OR CBT OR "cognitive therapy" OR "behavior therapy" OR mindfulness OR biofeedback OR neurofeedback OR "relaxation training" OR "autogenic training" OR meditation OR "mindfulness based treatmement") OR ab("Psychosocial treatment" OR psychotherapy OR "cognitive behavior treatment" OR CBT OR "cognitive therapy" OR "behavior therapy" OR mindfulness OR biofeedback OR neurofeedback OR "relaxation training" OR "autogenic training" OR meditation OR "mindfulness based treatmement")  #12 #1 OR #2  #13 #3 OR #4 OR #5 OR #6 OR #7 OR #8 OR #9 OR #10 OR # 11  #14 #12 AND #13 |
| --- |

**KoreaMed Search strategies**

| Clinical Trial, Comparative Study, Congresses, Controlled Clinical Trial, Evaluation Studies, Meta-Analysis, Multicenter Study, Observational Study, Original Article, Practice Guideline, Published Erratum, Randomized Controlled Trial, Retracted Publication, Retraction of Publication, Review, Twin Study, Validation Studies, Humans  ( 1946:2018 [DP] ) AND ( "Clinical Trial" [PT] OR "Comparative Study" [PT] OR "Congresses" [PT] OR "Controlled Clinical Trial" [PT] OR "Evaluation Studies" [PT] OR "Meta-Analysis" [PT] OR "Multicenter Study" [PT] OR "Observational Study" [PT] OR "Journal Article" [PT] OR "Original Article" [PT] OR "Practice Guideline" [PT] OR "Published Erratum" [PT] OR "Randomized Controlled Trial" [PT] OR "Retracted Publication" [PT] OR "Retraction of Publication" [PT] OR "Review" [PT] OR "Twin Study" [PT] OR "Validation Studies" [PT] ) AND ( "Humans" [MH] )  "headache" [mh]  "headache disorders" [mh]  "headache* [tiab] or migraine* [tiab]  "headache disorders, primary" [mh]  "migraine disorders" [mh]  "tension-type headache" [mh]  ( headache* [all] ) and ( ( tension [all] ) or ( "tension type" [all] ) or ( "muscle contraction" [all] ) or ( psychomyogenic [all] ) or ( stress [all] ) or ( ordinary [all] ) or ( essential [all] ) or ( idiopathic [all] ) or ( psychogenic [all] ) )  ( ( chronic [TI] OR chronic [AB] ) and ( daily [TI] OR daily [AB] ) and ( headache* [TI] OR headache* [AB] ) ) or ( ( daily [TI] OR daily [AB] ) and ( persistent [TI] OR persistent [AB] ) and ( headache* [TI] OR headache* [AB] ) )  "cluster headache" [mh]  ( ( cluster [all] ) and ( headache* [all] ) )  (neuralgi* [ALL])  menstrua* [tiab]  ( ( rebound [TI] OR rebound [AB] ) or ( transformed [TI] OR transformed [AB] ) ) and ( ( headache* [TI] OR headache* [AB] ) or ( migrain* [TI] OR migrain* [AB] ) )  ( ( medication [TI] or medication [AB] ) or ( drug [TI] or drug [AB] ) or ( ( pain [TI] and killer* [TI] ) or ( pain [AB] and killer* [AB] ) ) or ( analges* [TI] or analges* [AB] ) or ( triptan* [TI] or triptan* [AB] ) or ( apioid [TI] or apioid [AB] ) or ( caffeine [TI] or caffeine [AB] ) ) and ( ( ( over [TI] and use [TI] ) or ( over [AB] and use [AB] ) ) or ( ( mis [TI] and use [TI] ) or ( mis [AB] and use [AB] ) ) or ( associated [TI] or associated [AB] ) or ( induced [TI] or induced [AB] ) or ( abuse [TI] or abuse [AB] ) ) and ( headache* [TI] OR headache* [AB] or migrain* [TI] OR migrain* [AB] )  "Self Care" [mh] or "Social Support" [mh] or "Counseling" [mh]  "Self-Help Groups" [mh] or "Patient Participation" [mh]  "health education" [mh] or "consumer health information" [mh] or "patient education as topic" [mh] or "Communication" [mh] or "Health Communication" [mh]  "patient education handout" [mh]  "teaching" [mh] or " Programmed Instruction as Topic" [mh]  "communications media" [mh] or "Hotlines" [mh] or "Internet" [mh]  "information centers"[mh] or "information services"[mh] or "learning"[mh]  "Information Dissemination" [mh] or "Health Knowledge, Attitudes, Practice" [mh]  ( self care [TI] OR self care [AB] ) or ( "self-care" [TI] or "self-care" [AB] ) or ( selfcare [TI] OR selfcare [AB] ) or ( selfhelp [TI] OR selfhelp [AB] ) or ( "self-help" [TI] or "self-help" [AB] ) or ( "self help" [TI] OR "self help" [AB] ) or ( "self-management" [TI] OR "self-management" [AB] ) or ( "self management" [TI] OR "self management" [AB] )  ("social support" [tiab]) or ("support group*" [tiab])  ((education* [tiab]) or (learn* [tiab]) or (training [tiab]) or (teach* [tiab])) and ((program* [tiab]) or (patient* [tiab]) or (consumer* [tiab]) or (material* [tiab]) or (resource* [tiab]) or (aid* [tiab]))  (information [tiab]) and ((resource* [tiab]) or (leaflet* [tiab]) or (pamphlet* [tiab]) or (handout* [tiab]))  (patient [tiab]) and ((information [tiab]) or (knowledge [tiab]) or (website* [tiab]))  (workshop* [tiab]) or (counseling [tiab]) or (counselling [tiab]) or (seminar* [tiab]) or ("discussion group*" [tiab])  (factsheet* [tiab]) or ("advice line*" [tiab]) or ("advice-line*" [tiab]) or ("help line*" [tiab]) or ("help-line*" [tiab]) or (helpline* [tiab])  "Cognitive Therapy" [MH] or "Biofeedback, Psychology" [MH] or "Feedback" [MH] or "Feedback, Psychological" [MH] or "Autogenic Training" [MH] or "Breathing Exercises" [MH] or "Relaxation Therapy" [MH] or "Muscle Relaxation" [MH] or "Relaxation" [MH] or "Meditation" [MH] or "Mind-Body Therapies" [MH] or "Mind-Body Relations, Metaphysical" [MH] or "Psychotherapy" [MH]  "Imagery (Psychotherapy)" [mh]  (cognitive [tiab]) and ((behavior [tiab]) or (behaviour [tiab])) and ((therap* [tiab]) or (treatment [tiab]) or (technique* [tiab]))  (neurofeedback [tiab]) or (biofeedback [tiab])  ((controlled [tiab]) or (paced [tiab]) or (therap* [tiab]) or (exercise* [tiab])) and (breathing [tiab])  (respirat* [tiab]) and ((training [tiab]) or (exercise* [tiab]) or (therap* [tiab]))  (CBT [tiab]) or (qigong[tiab])  (guided [tiab]) and ((imagery [tiab]) or (visuali* [tiab]))  (mindfulness [tiab]) or (meditation [tiab]) or ("attention* control training" [tiab])  ((finger [tiab]) or (hand [tiab])) and (warming [tiab])  (handwarming [tiab]) or (fingerwarming [tiab])  (relaxation [tiab]) and ((therap* [tiab]) or (training [tiab]))  (relaxation [tiab]) and ((muscle* [tiab]) or (progressive [tiab]) or (therap* [tiab]) or (exercis* [tiab]) or (technique* [tiab]) or (training [tiab])) |
| --- |

**KMBASE search strategy**

| (((((((([KEYWORD=두통] OR [ALL=편두통]) OR [ALL=긴장성 두통]) OR [ALL=긴장형 두통]) OR [ALL=군발 두통]) OR [ALL=군발성 두통]) OR [ALL=군발형 두통]) AND NOT [TITLE=증례]) AND NOT [TITLE=동물 실험]) |
| --- |
